# The effect of flexible low-dose GnRH antagonist on pregnancy outcome in the fresh embryo transfer cycle of IVF-ET: a randomized controlled trial

**DOI:** 10.1186/s12958-022-00927-0

**Published:** 2022-03-22

**Authors:** Liping Feng, Ruiqi Fan, Aifang Jiang, Junyi Jiang, Qian Wang, Yujun Sun, Pengyun Qiao, Chune Ren, Tingting Yang

**Affiliations:** Department of Reproductive Medicine, Affiliated Hospital of Weifang Medical University, Shandong Province, People’s Republic of China

**Keywords:** Flexible low-dose GnRH antagonist, IVF-ET, Clinical pregnancy rate, Premature LH rise, Cetrotide

## Abstract

**Objective:**

To explore the practicality and effectiveness of a flexible low-dose protocol in the fresh embryo transfer cycle: reducing the total amount of antagonist by increasing the interval between administrations of Cetrotide.

**Methods:**

A total of 211 patients with normal ovarian reserve who accepted GnRH-ant protocol for IVF-ET were selected, and they were randomized to the flexible low-dose antagonist group (test group, *n* = 101) or the conventional dose antagonist group (control group, *n* = 110). The initial dose of Cetrotide in the test group was 0.25 mg every other day, and then the dose was adjusted to 0.25 mg every day based on the subsequent luteinizing hormone (LH) levels. The dosage of Cetrotide in the control group was 0.25 mg per day. The primary outcome was the clinical pregnancy rate. Secondary outcomes included the incidence of premature LH rise, total dosage of Cetrotide, number of oocytes retrieved, number of fertilized oocytes, number of high-quality embryos, biochemical pregnancy rate and ongoing pregnancy rate.

**Results:**

There was no significant difference in the general condition of the two groups. There was no significant difference in the clinical pregnancy rate (51.49% vs. 48.18%, *p* = 0.632) or the incidence of premature LH rise (18.81% vs. 15.45%, *p* = 0.584) between the two groups. However, the amount of Cetrotide used in the test group was significantly lower than that in the conventional dose antagonist group (1.13 ± 0.41 vs. 1.61 ± 0.59 mg, *p* < 0.001).

**Conclusion:**

The flexible low-dose antagonist protocol and the conventional dose antagonist protocol were equally effective in people with a normal ovarian reserve in the fresh embryo transfer cycle of IVF-ET.

## Introduction

In recent years, with the continuous development of assisted reproductive technology (ART), the medication protocol for controlled ovarian hyperstimulation (COH) has been gradually improved. The most commonly used ovulation stimulation protocols are the GnRH agonist protocol and the gonadotropin releasing hormone antagonist protocol (GnRH-ant). It was reported that the rate of live birth remained the same regardless of agonist or antagonist, but the clinical pregnancy rate was lower in the antagonist protocol [1]. A basic medical study has shown that the antagonist protocol produced abnormal inflammatory factors, which affected the implantation of embryos, thus reducing the clinical pregnancy rate of antagonist protocol [2]. In addition, GnRH-ant could impact endometrial receptivity by decreasing the expression of Hoxa10 during the implantation window and disrupting the development of podocytes, thereby affected embryo implantation and reduced the clinical pregnancy rate of the antagonist protocol [3]. However, the specific mechanism still needs further study.

To reduce the amount of antagonist, several studies have proposed that antagonists could be added according to the patient’s individual hormone levels and follicular conditions. An RCT research has proposed a flexible antagonist protocol in which GnRH-ant was added when LH > 10 IU/L, the diameter of the dominant follicle ≥ 12 mm or serum E_2_ > 150 pg/ml instead of being added on the fifth or sixth stimulation day [4]. Some study suggested that after assessment by the reproductive physician, when there was a risk of OHSS, medroxyprogesterone should be used instead of the antagonist to prevent a premature LH surge and to reduce the total amount of antagonist dosage [5]. In addition, several studies have shown that during controlled ovarian stimulation, the LH levels could be used as an indicator to add antagonists. In patients with persistently low LH levels (LH < 4.0 IU/L), antagonists might not be needed [6]. In addition, a previous preliminary study determined that the low-dose GnRH antagonist ganirelix could prevent premature luteinization at a similar rate with the daily dose in women undergoing IVF/ICSI treatment. However, this study did not compare the implantation rate or the clinical pregnancy rate between the two protocols [7]. Recently, other researchers have proposed a new flexible low-dose antagonist protocol, in which the initial dose of GnRH-ant was 0.125 mg per day, and then the dosage of GnRH-ant was adjusted according to the serum LH level [8, 9]. However, there is no 0.125 mg GnRH-ant preparation on the market, so there is a question about how to store the excess drug.

In this prospective randomized controlled trial, we attempt to reduce the total amount of antagonist by increasing the interval of medication. Then, we explore whether a flexible low-dose antagonist is effective by determining the clinical pregnancy rate and other reproductive outcomes, which can provide a new approach to a more rational and personalized application of antagonists.

## Materials and methods

### Patients

**Fig. 1:**
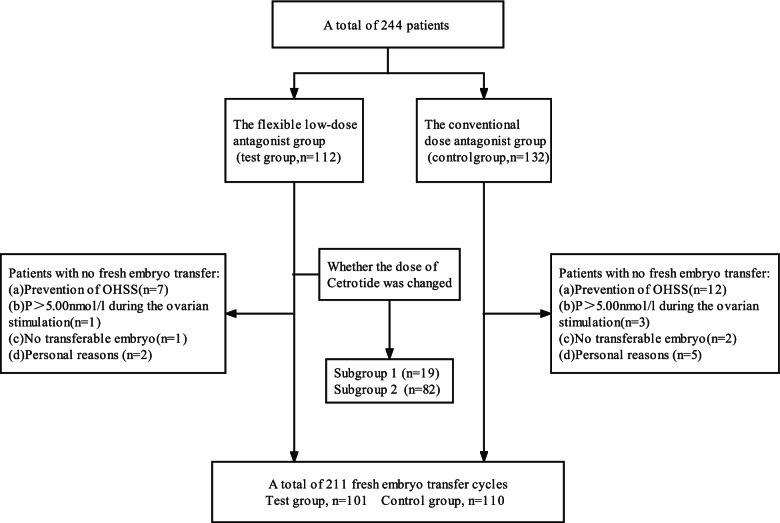
The flow chart of the trial

A total of 244 patients were selected, who accepted the GnRH-ant protocol for IVF-ET in the Department of Reproductive Medicine, Affiliated Hospital of Weifang Medical University. They were randomized to the test group (*n* = 112) and control group (*n* = 132) according to the random digital table method, every patient received a randomization number, which was calculated using a computer-generated program, then arranged all numbers from largest to smallest and divided into two groups. Since 33 patients did not complete the full protocol, 211 patients were included in the analysis, among which 101 patients were included in the test group and 110 patients were included in the control group. In the test group, the dose of GnRH-ant of 19 patients was adjusted according to the LH levels and 82 patients received consistently low doses. They were further divided into subgroup 1 and 2. The flow chart of the trial was shown in Fig. 1. The general information, ovarian stimulation and pregnancy outcome of the two groups were compared. All patients have been informed the aim of the study and signed the informed consent form before entering the study, which was approved by the Ethics Committee of the Affiliated Hospital of Weifang Medical University. All disease diagnosis and medication methods in this test were carried out in accordance with relevant regulations. This trial has been registered in the Chinese Clinical Trial Registry. Registration number: ChiCTR2000034834. (Registration date:21/07/2020, http://www.chictr.org.cn/index.aspx*).*

The inclusion criteria were as following: (a) 20–35 years old, (b) Anti-Mullerian hormone (AMH) ≥ 1.1 ng/ml, (c) basal FSH level < 10 IU/l, (d) no low ovarian response IVF-ET cycle, (e) using antagonist IVF and undergoing fresh embryo transfer [10]. The exclusion criteria included the following: (a) AMH < 1.1 ng/ml, (b) patients with untreated hydrosalpinx, (c) patients with endometrial polyps, (d) patients with untreated moderate to severe intrauterine adhesions, endometriosis and submucosal uterine fibroids, (e) spontaneous abortions ≥ 2 times or embryo transfer failure ≥ 2 times, (f) abnormal thyroid function or immune function, (g) chromosomal abnormality in either spouse requires pre-implantation embryo diagnosis, (h) contraindications related to the use of related drugs (such as moderate to severe liver and kidney damage and tumors of the ovary, breast, hypothalamus or pituitary gland).

### Ovarian stimulation

Flexible low-dose antagonist group (test group): urofollitropin (Gn, Lizhu, Zhuhai, Guangdong) was given on the third day of the menstrual cycle to initiate ovarian stimulation. We determined the starting dose from 150 to 225 IU according to age, AMH, antral follicle count (AFC), body mass index (BMI), and basal sex hormone levels. GnRH-ant Cetrorelix (Cetrotide, Merck Serono, Switzerland) was added from the sixth stimulation day. The initial dose of Cetrotide was 0.25 mg every other day, when the serum LH ≥ 10 IU/L or the serum LH level reached to 3 times of the baseline, the dose of Cetrotide was changed to 0.25 mg per day until the day of HCG. During the process of ovarian stimulation, we monitored the size of follicles and the levels of luteinizing hormone (LH), estradiol (E_2_) and progesterone (P), and adjusted the dosage of stimulation every 2 to 3 days. When there were two follicles reached a mean diameter of 18 mm or three follicles reached a mean diameter of 17 mm, 6000 IU human chorionic gonadotropin (HCG, Lizhu, Zhuhai, Guangdong) was used to induce ovulation. We performed oocyte retrieval 34–36 h after triggering under the guidance of transvaginal ultrasound.

Conventional dose antagonist group (control group): the timing and medication of ovarian stimulation were the same as those of the test group. The initial dose of Cetrotide was 0.25 mg every day until the day of HCG. Other follicle monitoring, hormone monitoring, and trigger timing were the same as the test group.

### Embryo transfer and pregnancy outcome judgment

IVF was performed on the day of oocyte retrieval. The luteal phase was supported from the first day after oocyte retrieval. We used 90 mg of sustained-release progesterone gel (Merck Serono, France) per day or dydrogesterone (Duffton, Abbott, The Netherlands) 10 mg twice a day [11]. Embryo transfer was performed under the guidance of transvaginal ultrasound on the third day after oocyte retrieval.

We graded the embryo morphology before transfer. High-quality embryos were defined as embryos with a cell count of 6–10 in the cleavage stage on day 3 and embryos with a morphological rating of I and II [12]. Then, we selected 1 or 2 embryos at the day 3 cleavage stage to transfer, except for under the following conditions: (a) the E_2_ on HCG day > 3500 pg/ml or the risk of OHSS was judged as high by the clinicians [13], (b) Gn days ≥ 15 days, (c) endometrial thickness > 14 mm or < 8 mm on the HCG day [14], (d) progesterone > 5 nmol/l during ovarian stimulation [15], (e) no transferable embryo, (f) personal reasons. When the cycles were at risk of OHSS, the fresh embryo transfer was cancelled, and the embryos were frozen.

All patients routinely continued corpus luteum support after embryo transfer. Serum β-HCG was tested at 14 days after embryo transfer, and β-HCG ≥ 5 IU/L was defined as biochemical pregnancy. Vaginal B-ultrasound was performed at 24–30 days after embryo transfer. The primary outcome was the clinical pregnancy rate, which was defined as the presence of a gestational sac identified by ultrasound examination, while a multiple pregnancy was defined as the presence of ≥ 2 gestational sacs. If the patient was pregnant after embryo transfer, luteal support was routinely given for 70 days after embryo transfer. Secondary outcomes included the incidence of premature LH rise, total dosage of Cetrotide, number of oocytes retrieved, number of fertilized oocytes, number of high-quality embryos, the biochemical pregnancy rate and the ongoing pregnancy rate.

### Sample size calculation

The primary hypothesis of this study was that the clinical pregnancy rate in test group was similar to control group. On the basis of actual data on the patients who accepted GnRH-ant protocol for IVF-ET in our hospital, we assume that after IVF-ET the proportions of clinical pregnancy are 45% per fresh cycle [9, 16]. With an effect size of 30%, power of 80% and two-sided α = 0.05, we would need 143 patients in total (the ratio between groups would be 1:1). Taking onside ration of dropout rate as 10% (such as canceling fresh embryo transfer), each group would include 80 participants (a total of 160 patients).

### Statistical analysis

SPSS 20.0 was used for data statistics and analysis. Measurement data was expressed as mean ± standard deviation, and the independent-sample test was used to analyze the difference in groups. Count data was expressed by rate, and comparison between groups was performed by chi-square test or Fisher's exact probability method. Two-sided test was used, and *p* < 0.05 was considered as statistically significant. Multiple regression analysis was used to analyze the factors that may affect the premature LH rise between the two subgroups.

## Results

A total of 211 patients were enrolled, among which 101 patients accepted a flexible low-dose GnRH-ant protocol (test group), and 110 patients accepted a conventional dose GnRH-ant protocol (control group). The basic statistics of the two groups of patients were shown in Table 1. There was no significant difference in the general information between the two groups (*p* > 0.05).

**Table 1:** Comparison of general information of the two groups of patients

	Test group	Control group	*P*
	(*n* = 101)	(*n* = 110)	
Age (years)	30.45 ± 3.09	30.94 ± 3.26	0.264
Infertility years (years)	3.29 ± 2.07	3.23 ± 2.12	0.836
BMI (kg/m^2^)	25.52 ± 4.21	25.08 ± 4.12	0.441
Antral follicle count (AFC)	17.82 ± 7.42	17.98 ± 9.23	0.889
Basal FSH level (IU/L)	6.59 ± 1.88	6.79 ± 1.84	0.453
Basal LH level (IU/L)	5.28 ± 3.06	5.45 ± 3.04	0.679
Basal E_2_ level (pg/ml)	44.61 ± 16.55	44.50 ± 17.37	0.962
Basal T level (μg/l)	0.26 ± 0.11	0.28 ± 0.15	0.273
PRL (ng/ml)	11.59 ± 7.16	11.91 ± 5.76	0.719
AMH (ng/ml)	4.34 ± 2.82	4.19 ± 3.13	0.701

The ovarian stimulation situation of the two groups was shown in Table 2. The E_2_, LH and P level of the test group on the start day of Cetrotide was lower than that of the control group, but there was no significant difference. In addition, there was also no significant difference in the incidence of premature LH rise between the two groups (18.81% vs. 15.45%, *p* = 0.584). The total dosage of Cetrotide in the test group was significantly less than that in the control group (1.13 ± 0.41 vs. 1.61 ± 0.59 mg, *p* < 0.001). There was no significant difference in the total amount of Gn, the total days of Gn used, number of oocytes retrieved, the number of fertilized oocytes, the number of embryos, and the available embryos in the two groups.

**Table 2:** The situation of ovarian stimulation in the two groups

	Test group	Control group	*p*
	(*n* = 101)	(*n* = 110)	
E_2_ on start day on Cetrotide (pg/ml)	371.00 ± 322.42	378.94 ± 351.71	0.865
LH on start day on Cetrotide (IU/L)	2.59 ± 2.00	2.95 ± 2.83	0.294
P on start day on Cetrotide (nmol/l)	1.01 ± 0.44	1.03 ± 0.40	0.691
E_2_ on HCG day (pg/ml)	2840.17 ± 1505.21	2629.78 ± 1580.56	0.324
LH on HCG day (IU/L)	3.93 ± 3.77	3.57 ± 3.13	0.452
P on HCG day(nmol/l)	2.81 ± 1.17	2.85 ± 1.03	0.777
Endometrial thickness on HCG day (mm)	11.26 ± 2.01	11.35 ± 2.28	0.779
Total dosage of Cetrotide (mg)	1.13 ± 0.41	1.61 ± 0.59	< 0.001
The day of Cetrotide (days)	6.75 ± 2.11	6.61 ± 2.29	0.638
Total dosage of Gn(IU)	1892.08 ± 475.08	1945.00 ± 507.80	0.436
The day of Gn(days)	10.31 ± 2.19	10.57 ± 2.35	0.398
Number of oocytes retrieved	12.93 ± 7.04	12.06 ± 6.54	0.355
Number of fertilized oocytes	9.71 ± 5.58	8.88 ± 4.98	0.254
Number of embryos	9.40 ± 5.55	8.75 ± 4.97	0.370
Number of available embryos	7.45 ± 4.82	6.93 ± 4.31	0.411
Number of high-quality embryos	3.89 ± 3.16	4.15 ± 2.91	0.543
Premature LH rise	18.81% (19/101)	15.45% (17/110)	0.584

At the beginning of the trial, a total of 244 patients were selected, among which 33 patients did not complete the full protocol. The reasons for the cycle cancellation were shown in Table 3. There was no significant difference in cycle cancellation rate between the two groups (9.82% vs. 16.67%, *p* = 0.136). No cycle was canceled because of preovulation. It should be noted that we did not compare the incidence of OHSS between groups in this trial. For that when patients were at risk of OHSS, fresh cycle embryo transfer would be cancelled and the embryos would be frozen. The number of cycles canceled due to prevention of OHSS did not significantly differ between the two groups (63.64% vs. 54.55%, *p* = 0.719).

**Table 3:** Reasons for Periodic Cancellation

	Test group	Control group	*p*
	(*n* = 11)	(*n* = 22)	
Prevention of OHSS	7/11 (63.64%)	12/22 (54.55%)	0.719
*P* > 5.00 nmol/l during the ovarian stimulation	1/11 (9.09%)	3/22 (13.64%)	1.000
No transferable embryos formed	1/11 (9.09%)	2/22 (9.09%)	1.000
Individual patient requests	2/11 (18.18%)	5/22 (22.73%)	1.000

As shown in Table 4, a total of 52 patients achieved clinical pregnancy in the test group (51.49%), while 53 patients in the control group achieved clinical pregnancy (48.18%). However, there was no significant difference in clinical pregnancy rate (*p* = 0.632). There was also no significant difference in implantation rate, biochemical pregnancy rate, ongoing pregnancy rate, ectopic pregnancy rate, multiple pregnancy rate and early abortion rate in the two groups (*p* > 0.05).

**Table 4:** The pregnancy outcomes of the two groups

	Test group	Control group	***p***
	(*n* = 101)	(*n* = 110)	
Clinical pregnancy rate	52/101(51.49%)	53/110(48.18%)	0.632
Embryos implanting rate	76/185(41.08%)	68/189(35.98%)	0.311
Biochemical pregnancy rate	56/101(55.45%)	60/110(54.55%)	0.896
Ongoing pregnancy rate	43/101(42.57%)	43/110(39.09%)	0.607
Ectopic pregnancy rate	3/101(2.97%)	2/110(1.82%)	0.672
Multiple pregnancy rate	9/101(8.91%)	11/110(10.00%)	0.818
Early abortion rate	6/101(5.94%)	8/110(7.27%)	0.786

A total of 19 patients in the test group had premature LH rise. After the occurrence of the LH rise, the dose of Cetrotide was changed to 0.25 mg per day until HCG day. According to whether the dose of Cetrotide was changed, the test group was divided into subgroup 1 (the amount of Cetrotide modified, *n* = 19) and subgroup 2 (the amount of Cetrotide not modified, *n* = 82). The general conditions and ovarian stimulation statistics were showed in Table 5. The age of subgroup 1 was significantly higher than that of subgroup 2. There was no significant difference in years of infertility, BMI, the levels of basal FSH or basal E_2_ between the two subgroups. At the same time, the basal LH, AMH and AFC of subgroup 1 were significantly higher than those of subgroup 2 (*p* < 0.05). Multiple regression was used to analyze which parameter in Table 5 had significant difference to influence the occurrence of premature LH rise between the two subgroups. As showed in Table 6, AFC was positively correlated with the incidence of premature LH rise (*p* < 0.05).

**Table 5:** Comparison of general conditions and pregnancy outcomes between subgroup 1 and subgroup 2

	Subgroup 1	Subgroup 2	***p***
	(*n* = 19)	(*n* = 82)	
Age (years)	31.79 ± 2.10	30.13 ± 3.21	0.035
Infertility years (years)	3.45 ± 1.63	3.25 ± 2.17	0.710
BMI(kg/m^2^)	26.53 ± 4.81	25.28 ± 4.06	0.248
Basal FSH level (IU/L)	6.49 ± 1.76	6.62 ± 1.92	0.788
Basal LH level (IU/L)	6.85 ± 2.79	4.91 ± 3.02	0.012
Basal E_2_ level (pg/ml)	49.71 ± 19.98	43.42 ± 15.56	0.136
Basal T level (μg/l)	0.29 ± 0.13	0.25 ± 0.10	0.176
Basal PRL level (ng/ml)	10.61 ± 6.08	11.81 ± 7.40	0.512
AMH (ng/ml)	6.24 ± 3.24	3.91 ± 2.54	0.001
AFC	22.79 ± 6.32	16.67 ± 7.20	0.001
E_2_ on start day on Cetrotide (pg/ml)	323.92 ± 240.52	381.91 ± 338.88	0.483
LH on start day on Cetrotide (IU/L)	2.49 ± 1.96	2.61 ± 2.02	0.808
P on start day on Cetrotide (nmol/l)	1.04 ± 0.61	1.00 ± 0.39	0.692
Endometrium on HCG day (mm))	12.21 ± 1.36	11.04 ± 2.08	0.004
E_2_ on HCG day (pg/ml)	3141.32 ± 1519.12	2770.39 ± 1502.69	0.336
LH on HCG day (IU/L)	8.27 ± 5.48	2.92 ± 2.32	0.001
P on HCG day (nmol/l)	3.19 ± 1.43	2.72 ± 1.10	0.119
Number of oocytes retrieved	12.32 ± 5.91	13.07 ± 7.30	0.675
Number of fertilized oocytes	9.47 ± 5.59	9.77 ± 5.61	0.837
Number of embryos	9.42 ± 5.57	9.39 ± 5.58	0.983
Number of available embryos	7.53 ± 5.06	7.43 ± 4.79	0.936
Number of high-quality embryos	3.84 ± 3.17	3.90 ± 3.18	0.941
Total day of Cetrotide	7.95 ± 2.57	6.48 ± 1.90	0.028
Total dosage of Cetrotide (mg)	1.32 ± 0.51	1.09 ± 0.37	0.025
Total day of Gn	11.37 ± 2.63	10.06 ± 2.02	0.019
Total dosage of Gn (IU)	2036.84 ± 502.04	1858.54 ± 465.38	0.141

**Table 6:** Multiple regression analysis on factors affecting premature LH rise

Item	Regression coefficient(B)	S. E	Wald	Degree of tree(df)	p(Sig.)	Exp(B)
Age (years)	0.180	0.127	2.014	1	0.156	1.197
Basal FSH level (IU/L)	-0.173	0.230	0.569	1	0.451	0.841
Basal LH level (IU/L)	0.124	0.112	1.219	1	0.194	1.135
AMH (ng/ml)	0.023	0.131	0.031	1	0.269	1.132
AFC	0.159	0.072	4.943	1	0.026	1.173

## Discussion

This is a new flexible low-dose antagonist protocol. In this prospective controlled trial, we tested this new flexible low-dose antagonist protocol intended to reduce the total amount of antagonist by increasing the interval between medications. The results of this study showed that the flexible low-dose antagonist protocol was not detrimental to clinical outcome compared to the conventional dose antagonist protocol. There was also no difference in the incidence of premature LH rise between the two groups. It was feasible and effective to reduce the GnRH-ant dose to 0.25 mg every other day, and then adjust the dose to 0.25 mg every day based on subsequent LH levels. The flexible low-dose antagonist protocol could reduce the amount of Cetrotide, reduce the number of injections, and reduce the economic burden on the patients. This flexible low-dose antagonist protocol may provide new approaches to the individualized application of antagonist protocols.

Embryo implantation is a complex process. This stage requires high-quality embryos, sufficient levels of progesterone, and an endometrial environment that is synchronized with the development of the embryo [17]. Some studies have reported that with an increase in the amount of antagonist, the implantation rate and pregnancy rate were reduced [18]. However, an RCT research showed that GnRH antagonist administration during the proliferative phase did not affect endometrial receptivity. It also reported that the embryo implantation rate and pregnancy rate were not different comparing those of the control group [19]. Other studies showed that the numbers of uterine natural killer cell and perforin expression levels were both increased in endometrium of GnRH-ant-treated patients, suggesting that GnRH-ant might reduce the receptivity of the endometrium [20]. In this study, the clinical pregnancy rate of the two groups was similar, although the total dosage of Cetrotide in the flexible low-dose antagonist protocol was significantly lower than that in conventional dose antagonist group. This may be because the reduction in Cetrotide dose was not sufficient to significantly increase the implantation rate. We found the flexible low-dose antagonist protocol did not increase the incidence of premature LH rise and progesterone level before oocyte retrieval, which might lead to the same effect between the two groups.

In IVF cycles, GnRH antagonists was unable to block the positive-feedback effect, suggesting that the clinical efficacy of GnRH antagonists was determined by the hyperstimulation process [21]. A previous preliminary study determined that alternate day administration of GnRH antagonist ganirelix could prevent premature luteinization to a similar extentwith the daily dose in women undergoing IVF/ICSI treatment, but they did not compare the pregnancy outcomes [7].The elimination half-life of ganirelix was about 13 h, but Cetrotide was about 30 h. In the trial powered to evaluate clinical pregnancy rate, we also should address whether alternate day was as effective as daily dose of Cetrotide in preventing premature LH rise. In this trial, there were 19 cases of premature LH rise in the test group and 17 in the control group. There was no significant difference in the incidence of premature LH rise between the two groups, which showed that the flexible low-dose antagonist protocol did not increase the risk of premature LH rise. The physiological LH concentration range is 0.5–10.0 IU/L [22]. A study in eight normally cycling women suggested that termination of the endogenous LH surge was related to ovarian factors rather than exhaustion of pituitary reserve [23]. Gonadotrophin surge-attenuating factor (GnSAF) is a nonsteroidal ovarian substance, which was mainly produced by small growing follicles. This factor played an important role in the control of the LH surge particularly during ovarian stimulation, when its bioactivity increases markedly [24, 25]. The positive feedback acts on the pituitary, leading to a premature LH surge [26]. The criteria for judging an early LH surge are not uniform at present, some researchers have indicated that a premature LH rise is defined as an LH level > 10 IU/L and a premature LH surge is defined as an LH level > 15 IU/L [27]. In this study, we referred to the basic LH level and defined a premature LH rise as an LH level ≥ 10 IU/L or an LH level exceeding the base level by 3 times. A premature LH surge would cause the quality of the follicle to decrease, reduce the pregnancy rate, and even lead to early follicle ovulation, leading to the cancellation of the cycle [28].

The flexible low-dose antagonist protocol did not have harmful effects on increasing the serum progesterone level before oocyte retrieval. The increase in serum progesterone could affect the expression of genes related to endometrial receptivity, thereby affecting embryo implantation [29]. Another studies had reported that the increase of serum progesterone in the late follicular phase would not only affect embryo quality [30, 31], but also reduce implantation rate and clinical pregnancy rate [32–34]. Additionally, there were also some studies showing that the increase in serum progesterone levels in the late follicular phase had no effect on embryo quality and cumulative live birth rate [35]. The results of this study showed that among the 211 patients transplanted in a fresh cycle, the serum progesterone level on HCG day in the test group was similar to that in the control group. The biochemical pregnancy rate and clinical pregnancy rate were same in the two groups. On the other hand, another study reported that the level of progesterone in serum on HCG day was positively correlated with the number of oocytes retrieved, and did not affect the quality of oocytes and embryos [36].

This study has some limitations. This study did not group the patients by age. Another study has shown that age can be an independent factor predicting pregnancy outcome [37]. However, the age of the patients in this study was ≤ 35 years and AMH ≥ 1.1 ng/ml, which could also indicate from another aspect that the ovarian reserve of these patients was normal. This study also did not evaluate the quality of the semen. There has been research showing that sperm DNA fragments could effectively predict pregnancy outcomes [38]. For the above reasons, the results of this study should be considered as a preliminary.

## Conclusion

In conclusion, the flexible low-dose antagonist protocol and the conventional dose antagonist protocol were equally effective in people with a normal ovarian reserve in the fresh embryo transfer cycle of IVF-ET.

## Data Availability

The datasets used and/or analyzed during the current study are available from the corresponding author on reasonable request.

## Abbreviations

IVF –ET: In Vitro Fertilization—Embryo Transfer
GnRH: Gonadotropin-Releasing Hormone
GnRH-a: Gonadotropin-Releasing Hormone Agonist
GnRH-ant: Gonadotropin-Releasing Hormone Antagonist
COH: Controlled Ovarian Hyperstimulation
ART: Assisted Reproductive Techniques
FSH: Follicle-Stimulating Hormone
LH: Luteinizing Hormone
P: Progesterone
E_2_: Estradiol
AFC: Antral Follicle Count
AMH: Anti-Mullerian Hormone
OHSS: Ovarian Hyperstimulation Syndrome
BMI: Body Mass Index
AI: Artificial Insemination
PCOS: Polycystic Ovary Syndrome
